# Robust therapeutic effects on COVID-19 of novel small molecules: Alleviation of SARS-CoV-2 S protein induction of ACE2/TMPRSS2, NOX2/ROS, and MCP-1

**DOI:** 10.3389/fcvm.2022.957340

**Published:** 2022-09-15

**Authors:** Ji Youn Youn, Jian Wang, Qian Li, Kai Huang, Hua Cai

**Affiliations:** ^1^Division of Molecular Medicine, Department of Anesthesiology, David Geffen School of Medicine at University of California, Los Angeles, Los Angeles, CA, United State; ^2^Division of Cardiology, Department of Medicine, David Geffen School of Medicine at University of California, Los Angeles, Los Angeles, CA, United States; ^3^Department of Cardiology, China-Japan Friendship Hospital, Beijing, China

**Keywords:** SARS-CoV-2, COVID-19, endothelial dysfunction, acute respiratory distress syndrome (ARDS), NADPH oxidase isoform 2 (NOX2)

## Abstract

While new variants of severe acute respiratory syndrome coronavirus 2 (SARS-CoV-2) constantly emerge to prolong the pandemic of COVID-19, robust and safe therapeutics are in urgent need. During the previous and ongoing fight against the pandemic in China, Traditional Chinese Medicine (TCM) has proven to be markedly effective in treating COVID-19. Among active ingredients of TCM recipes, small molecules such as quercetin, glabridin, gallic acid, and chrysoeriol have been predicted to target viral receptor angiotensin-converting enzyme 2 (ACE2) *via* system pharmacology/molecular docking/visualization analyses. Of note, endothelial dysfunction induced by oxidative stress and inflammation represents a critical mediator of acute respiratory distress syndrome (ARDS) and multi-organ injuries in patients with COVID-19. Hence, in the present study, we examined whether quercetin, glabridin, gallic acide and chrysoeriol regulate viral receptors of ACE2 and transmembrane serine protease 2 (TMPRSS2), redox modulator NADPH oxidase isoform 2 (NOX2), and inflammatory protein of monocyte chemoattractant protein-1 (MCP-1) in endothelial cells to mediate therapeutic protection against COVID-19. Indeed, quercetin, glabridin, gallic acide and chrysoeriol completely attenuated SARS-CoV-2 spike protein (S protein)-induced upregulation in ACE2 protein expression in endothelial cells. In addition, these small molecules abolished S protein upregulation of cleaved/active form of TMPRSS2, while native TMPRSS2 was not significantly regulated. Moreover, these small molecules completely abrogated S protein-induced upregulation in NOX2 protein expression, which resulted in alleviated superoxide production, confirming their preventive efficacies against S protein-induced oxidative stress in endothelial cells. In addition, treatment with these small molecules abolished S protein induction of MCP-1 expression. Collectively, our findings for the first time demonstrate that these novel small molecules may be used as novel and robust therapeutic options for the treatment of patients with COVID-19, *via* effective attenuation of S protein induction of endothelial oxidative stress and inflammation.

## Introduction

As on 1 May 2022, the cases of coronavirus disease 2019 (COVID-19) have surpassed 500 million with 6.0 million deaths worldwide (1). Since the outbreak of the COVID-19 pandemic, extensive research efforts have been made to reveal molecular mechanisms of severe acute respiratory syndrome coronavirus 2 (SARS-CoV-2) infection and the pathogenesis of COVID-19 for effective management of the disease, with many breakthroughs in areas such as rapid and specific identification of viral antigen and variants for accurate diagnosis, and development of robust vaccines and antiviral medications (2, 3). Nonetheless, the COVID-19 pandemic remains challenging to manage with the constant emergence of newly mutated SARS-CoV-2 variants (4). Besides previously defined variants of concern (VOC), Alpha (B.1.1.7), Beta (B.1.351), Gamma (P.1), and Delta (B.1.617.2), the newly emerged variant of SARS-CoV-2 Omicron (B.1.1.529), identified for the first time on 11 November 2021 (5) and now divided into three lineages (BA.1, BA.2, and BA.3) (6), have been known to possess a large number of mutations to confer higher transmissibility, and immune evasion against acquired immunity from natural infection, vaccination (breakthrough infections), or immunotherapies (7). It has been designated as a new VOC (8, 9). Therefore, robust therapeutics for COVID-19 remains in urgent need to contain the COVID-19 pandemic.

**FIGURE 1:**
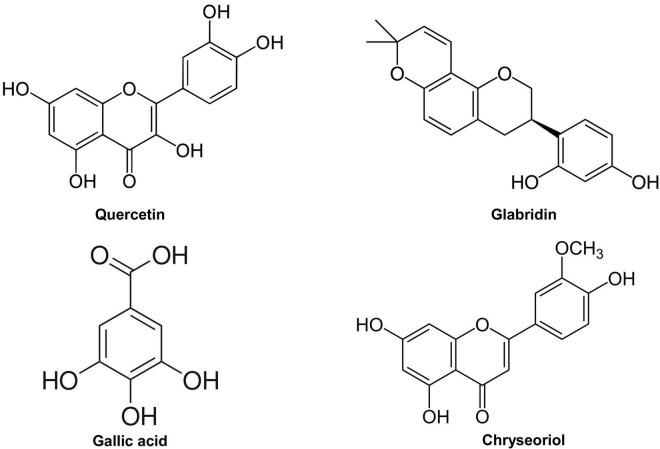
Chemical structures of quercetin, glabridin, gallic acid, and chrysoeriol.

During the previous and ongoing flight of the pandemic in China, accumulating evidence has shown that a combination of Traditional Chinese Medicine (TCM) and Western medicine is robustly effective in treating patients with COVID-19, including mild, moderate, and severe cases. Of note, early intervention with TCM has been shown especially beneficial in shortening the course of the disease and preventing the progression of the disease (10). Therefore, we have recently reviewed literature to identify active ingredients/small molecules of TCM recipes that had been predicted to target viral infection and consequent pathological events to effectively treat COVID-19 (11). Among them, quercetin, glabridin, gallic acid, and chrysoeriol (structures shown in Figure 1), four small molecules examined in the present study, were predicted to target viral receptor angiotensin-converting enzyme 2 (ACE2) *via* system pharmacology/molecular docking/visualization analyses, based on systematic reviews of literatures (11). Nonetheless, experimental and mechanistic data as to their effects on treating COVID-19 have been lacking. Of interest, some other natural compounds have also been tested or proposed for the treatment efficacies on COVID-19 (12–16).

Spike protein (S protein) of SARS-CoV-2 mediates fusion between viral envelope and cellular membranes for host cell entry through a series of events; first, S protein binds to the membrane-bound receptor of angiotensin-converting enzyme 2 (ACE2), the specific receptor for SARS-CoV-2; then, S protein is cleaved by furin into two subunits of S1 and S2; third, cleavage of S2 domain at discrete cleavage site S’ within S2 subunit by transmembrane serine protease 2 (TMPRSS2) exposes fusion peptide (FT) to be subsequently inserted into host cell membrane for fusion (17). Therefore, targeting ACE2 and/or TMPRSS2 to block initial viral entry into the cells could serve as an important therapeutic strategy for COVID-19.

Importantly, endothelial dysfunction is one of the key features of COVID-19, which is considered a major mediator of the development and progression of acute respiratory distress syndrome (ARDS) and multi-organ failure (18, 19, 23). Vascular pathologies, including endothelial morphological disruption, thrombosis, and perivascular inflammation, have been observed in the lungs of patients with COVID-19 (19, 20). Recent studies have shown that exposure of lung vascular endothelial cells to S protein or subunit alone triggers similar signaling responses as a live virus does upon binding to ACE2 receptor (21). Recombinant S1 subunit treated K-18 human ACE2 (hACE2) knock-in mice displayed features of acute lung injury and cytokine storm, as reflected by elevated cytokines in serum and increased expression of inflammatory cytokines/chemokines in the lung (22). We have recently shown that exposure to S protein or interleukin-6 (IL-6) (the primary mediator of cytokine storm) induces NADPH oxidase isoform 2 (NOX2)-dependent excessive oxidative stress in endothelial cells (23). S protein triggers upregulation of ACE2 in endothelial cells, and induction of proinflammatory protein monocyte chemoattractant protein-1 (MCP-1). All of these responses represent critical underlying mechanisms of endothelial dysfunction and vascular inflammation in COVID-19, driving the development of ARDS/multi-organ failure, and mortality.

Therefore, we examined in the present study whether the small molecules of quercetin, glabridin, gallic acid, and chrysoeriol, regulate ACE2, TMPRSS2, NOX2, and MCP-1 to mediate therapeutic protection against COVID-19. We followed previous experimental protocols in which we found estrogen administration is robustly effective in protecting against endothelial dysfunction *via* abrogation of ACE2 upregulation, and ACE2-dependent activation of NOX2 and MCP-1 (23). We found that quercetin, glabridin, gallic acid, or chrysoeriol treatment of endothelial cells attenuated viral S protein-induced upregulation in ACE2, active TMPRSS2, NOX2, MCP-1 and superoxide production, indicating therapeutic potential of these small molecule compounds for COVID-19 *via* preservation of endothelial function.

## Materials and methods

### Cell treatment

Bovine aortic endothelial cells (BAECs) (Genlantis, San Diego, CA, United States, passage 4 to 6) were cultured in M199 media supplemented with 10% fetal bovine serum (FBS), 1% vitamin, 1% L-glutamine, and penicillin-streptomycin as previously described (23–30). Confluent BAECs were starved in 5% FBS containing M199 media overnight before treatment with recombinant SARS-CoV-2 spike protein (S protein, 500 ng/ml, #10549-CV-100, R&D, Minneapolis, MN, United States) for 30 min. After 30 min of S protein treatment, cells were exposed to 100 nmol/l of quercetin (#Q4951, MilliporeSigma, St. Louis, MO, United States), glabridin (#G9548, MilliporeSigma, St. Louis, MO, United States), gallic acid (#G7384, MilliporeSigma, St. Louis, MO, United States), and chrysoeriol (derivative of Luteolin, #PHL85725, MilliporeSigma, St. Louis, MO, United States) for 24 h. The drugs were dissolved in dimethyl sulfoxide (DMSO) to make a stock solution and diluted for the treatment of cells. DMSO was also added to the control and S protein groups as vehicle control.

### Western blotting

After treatment, BAECs were lysed in lysis buffer (20 mmol/l Tris–HCl pH 7.4, 150 mmol/l NaCl, 1 mmol/l EDTA, 1 mmol/l EGTA, 2.5 mmol/l sodium pyrophosphate, 1 mmol/l β-glycerophosphate, 1 mmol/l sodium orthovanadate, and 1% Triton X-100, supplemented with protease inhibitor cocktail) for 20 min on ice and then supernatant separated by centrifugation at 12,000 rpm for 10 min at 4°C. Following protein concentration determination by DC protein assay (#5000112, Bio-Rad, Hercules, CA, United States), 25–40 μg of protein were separated in 10–15% sodium dodecyl sulfate-polyacrylamide gel electrophoresis (SDS-PAGE), followed by standard Western blotting protocol by probing with antibodies for ACE2 (1:1,000, #ab15348, Abcam, Waltham, MA, United States), TMPRSS2 (1:1,000, #ab92323, Abcam, Waltham, MA, United States), NOX2 (1:250, #611414, BD Biosciences, San Jose, CA, United States), MCP-1 (1:500, #ab9669, Abcam, Waltham, MA, United States), and β-actin (1:1,000, #A2066, MilliporeSigma, St. Louis, MO, United States) as we previously published (23). The protein bands were visualized by enhanced chemiluminescent methods, and band densities quantified using National Institutes of Health (NIH) Image J program.

### Determination of superoxide production by electron spin resonance

Superoxide production in BAECs was determined by electron spin resonance (ESR) (eScan, Bruker, Billerica, MA, United States) as we previously published (23–37). After treatment, cells were collected in cold modified Krebs/HEPES (KHB) buffer (99 mmol/l of NaCl, 4.69 mmol/l of KCl, 1.03 mmol/l of KH_2_PO_4_, 2.50 mmol/l of CaCl_2_, 1.20 mmol/l of MgSO_4_, 25.0 mmol/l of NaHCO_3_, 5.6 mmol/l of glucose, and 20.0 mmol/l of Na-HEPES, pH 7.35). The cell suspension was mixed with superoxide-specific spin trap CMH (1 mmol/l, #ALX-430-117-M250, Enzo Life Sciences, Farmingdale, NY, United States) in nitrogen gas bubbled KHB buffer containing diethyldithiocarbamic acid (5 μmol/l, #D3506, MilliporeSigma, St. Louis, MO, United States) and deferoxamine (25 μmol/l, #D9533, MilliporeSigma, St. Louis, MO, United States). Then, the cell mixture was loaded in glass capillaries and analyzed immediately by ESR. Superoxide signal was measured in the presence or absence of polyethylene glycol-superoxide dismutase (PEG-SOD) (20 U/ml, #S9549, MilliporeSigma, St. Louis, MO, United States). The PEG-SOD inhabitable superoxide signal was calculated and normalized to protein concentrations. The ESR settings used were: center field 3,480.00 G; sweep width 9.00 G; microwave frequency 9.79 GHz; microwave power 21.02 mW; modulation amplitude 2.47 G; 512 points of resolution; and receiver gain 1,000.

### Statistical analysis

All the data are presented as mean ± SEM. One-way ANOVA was used to compare the means among multiple groups with the Newman–Keuls *post hoc* test. *p* < 0.05 was considered statistically significant.

## Results

### Attenuation of severe acute respiratory syndrome coronavirus 2 spike protein-induced upregulation of angiotensin-converting enzyme 2/transmembrane serine protease 2 by small molecule compounds

**FIGURE 2:**
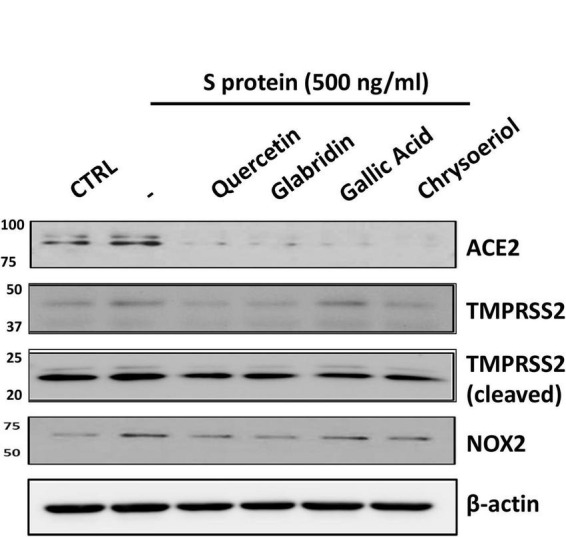
Representative Western blots of ACE2/TMPRSS2 and NOX2 in S protein and small molecules treated endothelial cells. Bovine aortic endothelial cells (BAECs) were pretreated with viral spike protein (S protein, 500 ng/ml) for 30 min before exposure to 100 nmol/l of small molecule compounds for 24 h. Shown are representative Western blots of ACE2, native/cleaved TMPRSS2 and NOX2 in S protein, and small molecules treated endothelial cells.

**FIGURE 3:**
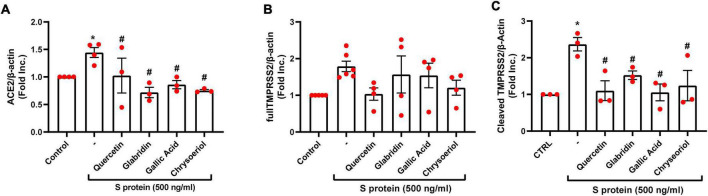
Attenuation of SARS-CoV-2 spike protein-induced upregulation of ACE2/TMPRSS2 by quercetin, glabridin, gallic acid, and chrysoeriol. Bovine aortic endothelial cells (BAECs) were pretreated with viral spike protein (S protein, 500 ng/ml) for 30 min before exposure to 100 nmol/l of small molecule compounds for 24 h. **(A)** Grouped data of ACE2 protein expression indicating that S protein-induced upregulation in ACE2 protein abundance was completely attenuated by small molecules. *n* = 3–4. **(B,C)** Grouped data of native and cleaved TMPRSS2 protein expression indicating that S protein activated proteolytic cleavage of TMPRSS2 was near completely attenuated by small molecules. Data are shown as mean ± SEM. *p < 0.05, **p < 0.01 vs. control group; #*p* < 0.05, ##*p* < 0.01 vs. S protein treated group by one-way ANOVA.

We have recently demonstrated that ACE2 is abundantly expressed in endothelial cells, and that viral spike protein (S protein) potently upregulates protein expression of ACE2 in both human and bovine endothelial cells (23). This response is fully reversible by estrogen treatment (23). Here, we examined the effects on endothelial ACE2 expression of novel small molecules of TCM recipes/ingredients found effective in treating COVID-19. The chemical structures of the small molecules are presented in Figure 1. As shown in Figures 2, 3A, quercetin, glabridin, gallic acid, or chrysoeriol (100 nmol/l) completely attenuated S protein-induced upregulation in ACE2 protein abundance in endothelial cells. Of note, these small molecules (100 nmol/l) also near completely alleviated S protein upregulation of cleaved/active form of TMPRSS2, while native TMPRSS2 was not significantly regulated (Figures 2, 3B,C). Following the binding of S protein to ACE2 *via* receptor-binding domain (RBD), TMPRSS2 cleaves the S2 domain to facilitate membrane fusion and viral entry. The cleaved active form of TMPRSS2 has been known to interact with ACE2 receptor (38). Hence, our results demonstrate potential therapeutic efficacies of TCM ingredient small molecules of quercetin, glabridin, gallic acid, and chrysoeriol, which are attributed to attenuation of ACE2 binding of S protein *via* downregulation of ACE2, and blockade of the activity of ACE2 via reduction of its interaction with the activated form of TMPRSS2.

### Attenuation of severe acute respiratory syndrome coronavirus 2 spike protein-induced upregulation of NADPH oxidase isoform 2 and excessive superoxide production by small molecule compounds

**FIGURE 4:**
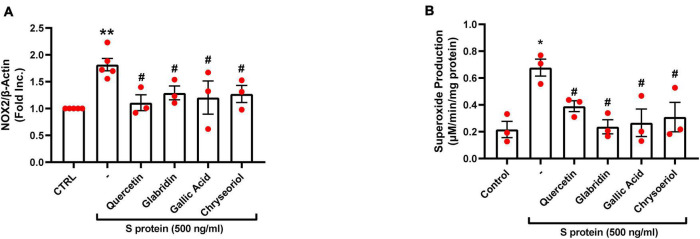
Attenuation of SARS-CoV-2 spike protein-induced upregulation of NOX2 and excessive superoxide production by quercetin, glabridin, gallic acid, and chrysoeriol. Bovine aortic endothelial cells (BAECs) were pretreated with viral spike protein (S protein, 500 ng/ml) for 30 min before exposure to 100 nmol/l of small molecule compounds for 24 h. **(A)** Grouped data of NOX2 protein expression indicating upregulation of NOX2 by S protein, which was near completely attenuated by small molecules. *n* = 3–5. **(B)** Grouped data of superoxide production as determined specifically and quantitatively by electron spin resonance (ESR), indicating S protein induction of excessive superoxide production in endothelial cells, which was near completely alleviated by small molecules. Data are shown as mean ± SEM. *n* = 3, **p < 0.01 vs. control group; #*p* < 0.05 vs. S protein treated group by one-way ANOVA.

We have recently shown that ACE2-dependent selective activation of NOX2 and ROS production by viral S protein can be completely reversed by estrogen (23). In the present study, we also examined the effects on NOX2 protein expression and endothelial superoxide production of novel small molecules of TCM recipes/ingredients found effective in treating COVID-19. As shown in Figures 2, 4A, the expression of NOX2 was upregulated by S protein, which is consistent with our previous findings (23). Treatment with quercetin, glabridin, gallic acid, or chrysoeriol following S protein stimulation of endothelial cells near completely attenuated NOX2 protein upregulation at a concentration of 100 nmol/l, indicating robust reversal effects of these small molecules on NOX2 activation. S protein stimulation markedly upregulated superoxide production in endothelial cells, which is consistent with our previous observations (23). Treatment with small molecules (100 nmol/l) following S protein stimulation of endothelial cells near completely alleviated increased superoxide production, indicating robust therapeutic potential of these small molecules on S protein-induced oxidative stress in endothelial cells (Figures 2, 4B).

### Attenuation of severe acute respiratory syndrome coronavirus 2 spike protein-induced upregulation of monocyte chemoattractant protein-1 by small molecule compounds

**FIGURE 5:**
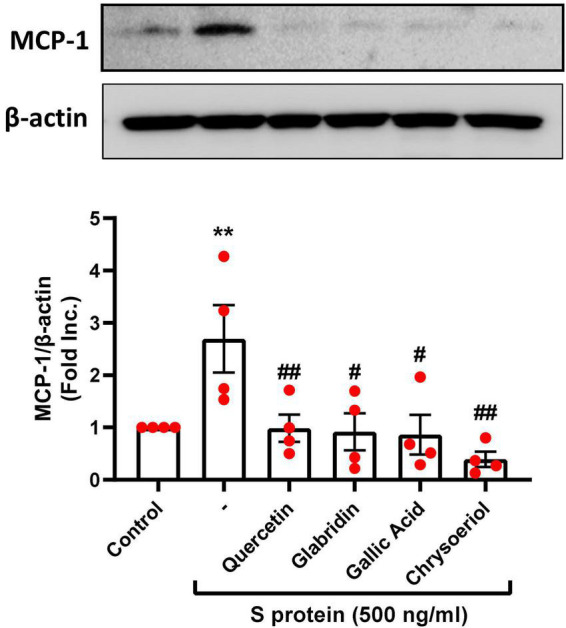
Attenuation of SARS-CoV-2 spike protein-induced upregulation of MCP-1 by quercetin, glabridin, gallic acid, and chrysoeriol. Bovine aortic endothelial cells (BAECs) were pretreated with viral spike protein (S protein, 500 ng/ml) for 30 min before exposure to 100 nmol/l of small molecule compounds for 24 h. Shown are representative Western blots and grouped data of MCP-1 protein expression indicating upregulation of proinflammatory protein MCP-1 by S protein, which was completely attenuated by small molecules. Data are shown as mean ± SEM. *n* = 4. **p < 0.01 vs. control group; #*p* < 0.05, ##*p* < 0.01 vs. S protein treated group by one-way ANOVA.

The proinflammatory cytokines such as MCP-1 were found to be elevated along with other inflammatory mediators in patients with COVID-19 (39). In the previous study, we found that S protein exposure induced upregulation of pro-inflammatory protein MCP-1, which was completely alleviated by estrogen (23). Interestingly, treatment with quercetin, glabridin, gallic acid, or chrysoeriol (100 nmol/l) also abolished S protein induction of MCP-1 (Figure 5). The alleviation in MCP-1 can prevent attraction of monocytes/macrophages to endothelial cells to eliminate hyperinflammatory state, as dysregulated levels of MCP-1, –2, and –3 were observed in plasma samples of hospitalized patients with COVID-19 (39). Therefore, quercetin, glabridin, gallic acid, or chrysoeriol (luteolin derivative) can effectively attenuate endothelial dysfunction and inflammation *via* inhibition of ACE2/TMPRSS2, NOX2-dependent superoxide production, and MCP-1, leading to abrogation of ARDS/multi-organ failure and mortality in patients with COVID-19.

## Discussion

Spike protein (S protein) of SARS-CoV-2 mediates host cell entry *via* angiotensin-converting enzyme 2 (ACE2) and transmembrane serine protease 2 (TMPRSS2), targeting of which could serve as an important therapeutic strategy for COVID-19. In the present study, we hypothesized that small molecules quercetin, glabridin, gallic acid, and chrysoeriol, derived from active ingredients of TCM recipes proven effective in treating COVID-19 (11), protect against COVID-19 by inhibiting viral receptors of ACE2 and TMPRSS2 on endothelial cells, thus regulating expression of NOX2 and MCP-1, as well as endothelial cell production of superoxide. We found that expression of ACE2 and cleaved/active form of TMPRSS2 were significantly upregulated by SARS-CoV-2 S protein, both of which were abolished or near completely attenuated by treatment with the small molecules described above. In addition, treatment of endothelial cells with quercetin, glabridin, gallic acid, or chrysoeriol near completely reversed S protein-induced NOX2 activation and NOX2-dependent production of superoxide, as well as upregulation of proinflammatory proteins such as MCP-1.

Among the four molecules, quercetin, glabridin, and chrysoeriol (luteolin derivative) are naturally occurring flavonoids, while gallic acid is a natural polyphenolic compound. Natural flavonoids have been known to reduce oxidative stress and inflammation in endothelial cells, so that it contributes to restoration of endothelial function in diabetes (40), though detailed molecular mechanisms have remained unclear. Gallic acid was demonstrated to have anti-obesity properties by suppressing lipogenesis, improving insulin signaling, and reducing proinflammatory responses and oxidative stress (41). Whether or not these small molecules regulate pathways such as NADPH oxidases has remained unclear. Of note, these small molecules have been predicted to target ACE2 by system pharmacology/molecular docking/visualization analyses. Here, we treated endothelial cells with these small molecules following initial exposure of the cells to S protein, to examine potential reversal effects on pathological consequences of these small molecules. Indeed, four small molecules investigated in this study completely attenuated S protein-induced upregulation in ACE2 protein abundance. Of note, these small molecules near completely attenuated S protein upregulation of cleaved/active form of TMPRSS2, while native TMPRSS2 was not significantly regulated. The detailed molecular mechanisms underlying the inhibition on ACE2 of these small molecules warrant further investigation, while the predicted binding of these small molecules to ACE2 by docking studies might indeed underlie ACE2 inhibition by these compounds (42, 43). Besides the direct targeting of ACE2, network pharmacology analyses predict that these molecules might modulate transcription factors (TFs), including hepatocyte nuclear factor 4 alpha (HNF4A) and peroxisome proliferator-activated receptor gamma (PPARG), or microRNAs (miRNAs), including hsa-miR-2113 and hsa-miR-421, resulting in modulation of these upstream events to target ACE2 (43).

For SARS-CoV-2 entry into host cells, ACE2 and TMPRSS2 are critically required (44). Of note, “viral entry inhibitors,” the antiviral class of drugs to block viral entry by inhibiting both TMPRSS2 and ACE2, have been actively investigated for the development of new drugs for COVID-19 (3, 44). The United States Food and Drug Administration (USFDA) has approved approximately 2,800 investigative molecules for further evaluations, which were selected using a virtual docking tool identifying dual-target inhibition of TMPRSS2 and ACE2 (44). In an *in silico* drug repurposing study, lopinavir and valrubicin have been shown superior in terms of dual inhibition (44). Compared to the antivirals targeting the viral life cycle to treat severe, hospitalized patients, such as remdesivir, viral entry inhibitors have the advantages for early intervention of COVID-19 (45). By blocking viral entry, these antivirals possess the potential to prevent disease transition into severe cases and shorten the disease course, which is in line with the observed benefits of TCM therapies during early intervention (10, 11, 45). Hence, as the active components of the proven TCM recipes, these small molecules might represent suitable candidates as viral entry inhibitors.

Oxidative stress and inflammation have been implicated in ARDS and multi-organ injuries in COVID-19 (18). We have recently shown that S protein stimulation of endothelial cells leads to marked elevation in intracellular superoxide production to induce endothelial dysfunction, which is also characterized by activation of inflammatory signaling, including production of cytokines and chemokines such as IL-6 (one of the key cytokines mediating cytokine storm during the pathogenesis of COVID-19) and MCP-1 (23). Our study has demonstrated that exposure to S protein or IL-6 induces excessive oxidative stress in endothelial cells, which is mediated specifically by activation of NADPH oxidase isoform 2 (NOX2), but not NOX1 or NOX4 (23). Here, we confirmed that S protein induced upregulation of ACE2 in endothelial cells, and ACE2-dependent activation of NOX2, superoxide production, and induction of proinflammatory protein MCP-1. All of these responses represent critical underlying mechanisms of endothelial dysfunction and vascular inflammation in COVID-19, driving the development and progression of ARDS/multi-organ failure, and mortality. In this study, four small molecules of quercetin, glabridin, gallic acid and chrysoeriol near completely attenuated S protein-induced upregulation of NOX2 protein expression, which subsequently abolished overproduction of superoxide, indicating their preventive action against S protein-induced oxidative stress in endothelial cells. In addition, S protein exposure resulted in induction of pro-inflammatory protein MCP-1, which was completely alleviated by treatment with these small molecules. Of note, MCP-1 was found upregulated by oxidative stress activation of nuclear factor-kappa B (NF-_K_B) in endothelial cells (46).

**FIGURE 6:**
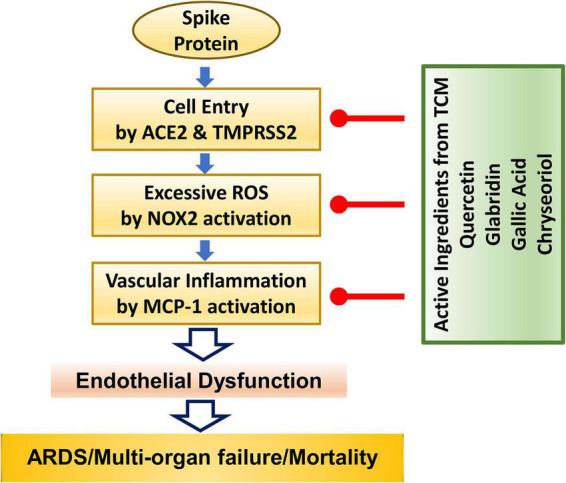
The anti-COVID-19 effects of small molecule compounds of quercetin, glabridin, gallic acid, and chrysoeriol. Summary of therapeutic actions of active small molecules in protecting against S protein-induced endothelial dysfunction and inflammation to result in attenuation of ARDS/multi-organ failure and mortality in patients with COVID-19.

In conclusion, we demonstrate for the first time that four small molecules of quercetin, glabridin, gallic acid and chrysoeriol may serve as novel and robust therapeutic options for COVID-19 by: (1) inhibiting viral entry into endothelial cells through downregulation of ACE2/TMPRSS2; (2) preserving endothelial function *via* attenuation of excessive superoxide production elicited by S protein activation of NOX2; and (3) abolishing endothelial inflammation by blocking S protein induction of pro-inflammatory protein MCP-1. These protective effects of the small molecules are anticipated to effectively alleviate ARDS/multi-organ failure/mortality *via* abrogation of endothelial dysfunction. The molecular pathways invovled are shown schematically in Figure 6. Therefore, these small molecules can be immediately repurposed as novel therapeutic options for the treatment of patients with COVID-19.

## Data availability statement

All data of this study are presented in the article.

## Ethics statement

Ethical review and approval was not required for this study in accordance with the local legislation and institutional requirements.

## Author contributions

JY: experimentation, data analysis, and drafting of the manuscript. JW, KH, and QL: drafting of the manuscript. HC: project design and administration, data analysis, drafting, and finalizing of the manuscript. All authors contributed to the article and approved the submitted version.
